# Fruit quality retention and shelf-life extension of papaya through organic coating

**DOI:** 10.1016/j.heliyon.2024.e41293

**Published:** 2024-12-16

**Authors:** Sazia Jahan, Joydeb Gomasta, Jahidul Hassan, Md Habibur Rahman, Md Abdul Kader, Emrul Kayesh

**Affiliations:** a Department of Horticulture, Bangabandhu Sheikh Mujibur Rahman Agricultural University, Gazipur, 1706, Bangladesh; b Department of Entomology, Bangabandhu Sheikh Mujibur Rahman Agricultural University, Gazipur, 1706, Bangladesh

**Keywords:** *Carica papaya* L., Climacteric fruit, Postharvest treatment, Quality parameters, Disease incidence, Storage life

## Abstract

Papaya (*Carica papaya* L.) is a climacteric fruit which lose quality and shelf life quickly due to physiological decay and microbial infection after harvest. The study was conducted to evaluate newly applied clybio formulation (0.2 %) along with the existing effective concentration of chitosan (1 %), aloevera gel (50 %), seaweed extract (1 %) and uncoated papaya (control) fruits on post-harvest physicochemical properties and disease incidence when stored at 25 ± 1 °C and 85–90 % relative humidity. Quality parameters were available up to 12 days of storage (DAS) for chitosan and clybio treated papaya where it was 9 DAS for aloevera gel and seaweed treated papaya and 6 DAS for control papaya. Before decay of all the coated papaya at 9 DAS, chitosan (1 %) performed superior in retaining maximum reducing sugar (0.77 %, 1.41 % and 3.85 % more than aloevera, seaweed and clybio application, respectively), β-carotene (10.94 %, 12.5 % and 9.89 % greater than aloevera, seaweed and clybio coatings, respectively), total flavonoids content (18.36 %, 29.81 % and 25.29 % better than aloevera, seaweed and clybio treatments, respectively), total antioxidant activity (21.85 %, 68.2 % and 47.91 % than noted in aloevera, seaweed and clybio formulations, respectively) and potassium content (3.14 % and 9.32 % than aloevera and clybio treatments, respectively). In addition, clybio gave better results over chitosan up to completion of shelf life (12 DAS) such as retention of ascorbic acid (6.21 %), non-reducing sugar (13.48 %), magnesium content (8.31 %) and disease incidence (20 %). Thus, besides preserving nutraceutical property, chitosan and clybio coated papaya remained edible for further 6 days compared to control, and 3 days over aloevera gel and seaweed extract treatment. These findings suggest the use of chitosan and clybio formulation for preserving quality parameters and extending the storage life of papaya.

## Highlights

• Clybio formulation (bacterial consortium) used for the first time in papaya.
• Among four organic coatings, chitosan and clybio formulation were found superior for disease reduction and shelf-life extension.
• Chitosan and clybio effectively retained functional and mineral properties of papaya.
• Disease incidence, ascorbic acid, TSS, β-carotene, sugar contents and minerals like Ca, K were the key correlated traits.
• Clybio and chitosan can be employed for short-term fruit preservation assuring edible quality retained.

## Graphical abstract

**Figure:**
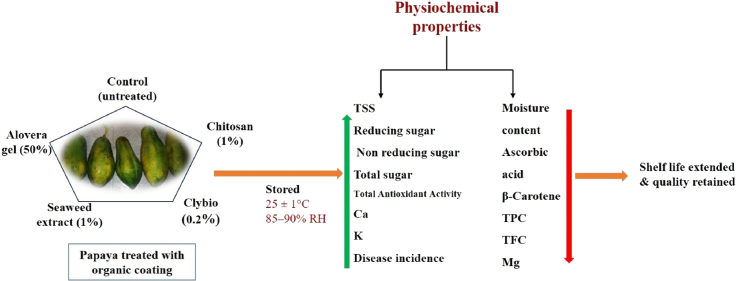

## 1 Introduction

Papaya (*Carica papaya* L.) is one of the important fruit crops having high nutritive and economic value grown in the world. The fruit is termed as the ‘wonder fruit of the tropics’ due to its high contents of vitamins, minerals, antioxidant and phytochemicals that have chemoprotective, antidiabetic, antibacterial, antiplasmodial and antifungal properties [1,2] However, being a climacteric fruit, papaya faces postharvest losses due to biotic (microbial) and abiotic stresses (oxidative stress, accelerate the internal biochemical processes of papaya metabolism) which lead a very short postharvest life [3,4]. It is remarkable that postharvest loss of papaya ranged from 25 to 30 % in Bangladesh that accounts for a considerable deficit of vitamins and minerals. Hence, researchers outlined a lot of ways including the use of appropriate post-harvest handling, packaging, transportation, and storage practices to minimize the amount of post-harvest loss [5]. Now a days, compounds derived from natural sources as an edible coating (e.g. carnauba, aloevera gel, seaweed, paraffin, shellac, beeswax and chitosan) and probiotics are therefore used during storage to extend shelf life, having minimal or negligible effects on human health [6,7].

This research has focused on minimizing postharvest losses and extending shelf life of papaya by different organic coating such as chitosan, aloevera gel, seaweed and clybio formulation. However, the first three materials have been used previously in papaya [3,8,9], clybio formulation used for the first time. Chitosan has chosen for its suitability in postharvest quality control from previous study used in papaya as well as other temperate and tropical fruits such as fig, banana and cherry tomato to prolong the storage life and control decay [10]. Chitosan treatment at postharvest could create a protective, semi-permeable film around the product that regulate gas exchange, reducing oxygen intake and carbon dioxide release, which could slow down the respiration rate to prevent spoilage [11]. In addition, chitosan limits oxidative stress, which would otherwise degrade essential components like vitamins (especially vitamin C), antioxidants (such as carotenoids), and other bioactive compounds [12]. Aloevera gel also used as preservative in papaya and some other fruits such as hog plam, duke cherries, gola guava etc. [13,14]. Aloe vera has inherent antimicrobial and antifungal properties that inhibit the growth of spoilage-causing pathogens, including bacteria and fungi. By preventing microbial contamination, it significantly reduces decay and enhances the fruit's overall storage quality [15]. Brown seaweeds used in agriculture upon last few decades as a fertilizer and coating agent due to its antibacterial and antifungal properties [16]. Moreover, the presence of polysaccharides and nutrients such as iodine and potassium in seaweed, can enhance the product's metabolism, improving its overall stress tolerance during storage [17]. The newly manufactured microbial formulation clybio already used in litchi as a postharvest treatment that gave positive results [18]. Clybio mainly contains three different micro-organisms, including yeast, *Bacillus* spp. and *Lactobacillus.* Yeast has biofilm formation capacity, cell wall degrading enzymatic activity, involvement in reducing oxidative stress and maintaining desired environment of surface wounds [19]. Lactic acid bacteria (*Lactobacillus*, *Enterococcus*) can keep the physicochemical and sensory qualities of some fresh-cut fruits such as jackfruit, pineapple and apples, and has no adverse effects [[20], [21], [22]]. Bacillus natto is a subspecies of *Bacillus subtilis* can suppress many pathogenic microorganisms, improve the protein quality of fruits and produce plenty of the key nutrients (vitamins, enzymes, carotenoids, lipids, etc.) [[23], [24], [25]]. Together, these three microorganisms can enhance the fruit's microbial stability, reduce decay, and improve preservation by producing natural antimicrobial compounds that protect fruits from spoilage. Therefore, the aim of this study was to evaluate the effect of newly applied clybio formulation with previously used chitosan, aloevera gel, and seaweed extract on maintaining postharvest quality and prolonging the shelf life of papaya during normal storage conditions.

## 2 Material and methods

### 2.1 Sample collection and experiment set up

Healthy and disease-free mature papaya (Local cultivar; *Badsha Rabi*) of color index 3 (yellowish green papaya) [26] were harvested and collected from a local garden of Sreepur, Gazipur (24.1996° N, 90.4807° E) to the Tissue Culture Laboratory, Department of Horticulture, Bangabandhu Sheikh Mujibur Rahman Agricultural University, Gazipur 1706, Bangladesh. After washing with distilled water and drying under air, treatments were applied on samples and stored at room temperature (RT) (25 ± 1 °C) and 85–90 % relative humidity (RH). According to the study goal, papaya fruits were treated with naturally derived chitosan (1 %), aloevera gel (50 %), seaweed extract (1 %) and clybio formulation (0.2 %) besides untreated papaya following completely randomized design (CRD) with three replications. Thus, the current experiment consisting of five treatments, each treatment replicating thrice, included a total of 150 papayas, 10 papayas per replication (5 papayas for evaluating disease and shelf life, another 5 for chemical analysis).

### 2.2 Treatment preparation and application

Low molecular weight chitosan (1526.464 g/mol) (Viscosity: 10–150 mPa s, Deacetylated degree: 90 %, Sisco Research Laboratories Pvt. Ltd, India) was used for treatment. For preparing 1 % solution, 1.0 g of chitosan dissolved in 100 mL distilled water containing 2 % acetic acid with pH being adjusted to 5.7 using 1N NaOH. Likely for preparing 50 % alovera gel solution, fresh aloevera was grounded in a blender with water (500 g/1000 mL) and resulting mixture was filtered to remove the fibers and was pasteurized at 70 °C for 45 min and cooled immediately for stabilization and pH adjusted at 4 by using ascorbic acid (2.0 g/L) and citric acid (4.5 g/L). The stability of gel was improved by using 1 % agar [27]. For preparing 1 % seaweed, 5 g dry seaweed *(Gracilaria tertuistipitata)* was immersed in 500 mL of water and boiled for 15 min at 100 °C. After cooling, it was filtered through Whatman filter paper No. 1 and allowed to set at room temperature (25 ± 2 °C) as proposed by Banu et al. [7]. To prepare 0.2 % clybio solution, 2 mL of clybio (Yeast fungus, *Bacillus natto* and *Lactobacillus*) was added in 1000 mL water. A total of 3 L treatment solution was prepared for each of the four treatments so that 1.0 L solution could be allocated per replication. The fruits were dipped in solution for 2 min and after that fruits were air dried in the laboratory.

### 2.3 Estimation of weight loss, total soluble solids, pH and sugar contents

#### 2.3.1 Weight loss and moisture content

Treated and untreated fruits were weighed at 3, 6, 9 and 12 days after storage and compared with the weight of freshly harvested papaya to estimate the weight loss and expressed as percentage of weight. Meanwhile, during storage the fruit samples were weighed before and after drying at every sampling day to measure moisture content as percentage following the formula (equation (1)) described by Awad et al. [28].

(1) $$\text{Moisture}\;\text{content}\;\left(\%\right)=\frac{\text{Initial}\;\text{weight}‐\text{Final}\;\text{weight}\;\text{after}\;\text{drying}}{\text{Initial}\;\text{weight}}\times 100$$

#### 2.3.2 Total soluble solid (TSS), pH, reducing sugar, non-reducing sugar and total sugar

Total soluble solids (TSS) was estimated by hand refractometer (Model: Atago N1, Japan) as percent Brix. The pH of the fruit juice was determined by using a digital pH meter (PHSJ-3F, iste.inc, Korea). While, reducing (mg/g), and total sugars (mg/g) were estimated through titration method as described by Somogyi [29] using Bertrand A, B and C solutions. Non-reducing sugar (mg/g) calculated from total and reducing sugars.

### 2.4 Determination of ascorbic acid and β-carotene

Ascorbic acid (mg/100 g) content was determined by titration method [30]. Exactly 20 g fruit was blended and centrifuged at 4000×*g* for 20 min. For ascorbic acid determination, 10 mL of the prepared fruit sample extract was taken in a 100 mL conical flask and 5 mL of 5 % potassium iodide (KI), 2 mL of glacial acetic acid were added and titrated with 0.001N potassium iodate (KIO_3_) solution.

Whereas, β-Carotene was estimated by spectrophotometric method described by Nagata and Yamashit [31]. Briefly, 1 g of fruit sample was crushed and mixed thoroughly with 10 mL of acetone: hexane (4:6) solution. After filtering the optical density (OD) of the supernatant was measured by spectrophotometer (Model no. 200-20, Hitachi, Japan) at 663 nm, 645m, 505 nm and 453 nm. Calculation was done by the following formula (Equation (2)).

(2) $$\beta -\text{carotene}\;\left(\text{mg}/100g\right)=0.216_{\left(\mathrm{OD}663\right)}+0.452_{\left(\mathrm{OD}453\right)}-1.22_{\left(\mathrm{OD}645\right)}-0.304_{\left(\mathrm{OD}505\right)}$$

### 2.5 Determination of phenols, flavonoids and antioxidant activity

The total phenol content (TPC) was determined according to Folin Cioucalteu (FC) method done by Khanam et al. [32] with some modification. About 0.5 mL of sample (1000 ppm) and gallic acid standard solutions were mixed with 2.5 mL of FC reagent (10 times diluted). Two min later the mixture was incubated for 30 min adding 2 mL of 7.5 % Na_2_CO_3_. Finally, the absorbance was read at 765 nm by UV–VIS spectrophotometer (Model 170-30, Hitachi, Japan) against blank and results were expressed as mg gallic acid equivalent (GAE) per 100 g sample in dry basis (DW). Meanwhile, aluminum chloride colorimetric method [33] was employed to determine the total flavonoid content. For sample extraction, 0.5 mg of dry sample was mixed with 5 mL of methanol and shaked in orbital shaker at 100 g for 2.5 h at room temperature and centrifuged by filter paper (Whatman Cat No 1001 125, size no 125 mm). Exactly, 3 mL of methanol, 0.2 mL of 10 % aluminum chloride (Al_2_Cl_3_), 0.2 mL of 1 M potassium acetate and 5.6 mL of distilled water was added in 1 mL of plant extracts and quercetin standards. Incubating for 30 min the absorbance of the samples was measured at 420 nm using spectrophotometer (Model 170-30, Hitachi, Japan) against blank and the results were expressed as mg quercetin equivalents (QE) per 100 g of dry weight of the sample (DW). Whereas, the total antioxidant activity was determined using 2,2-diphenyl-1- picrylhydrazyl (DPPH) assay [34] with slight modification. Rightly, 1 mL of each plant extract and ascorbic acid standards was taken in test tubes, 3 mL of 1 M DPPH was added and made volume up to 10 mL with 99 % methanol. Rest them 30 min and took reading at 517 nm against blank by spectrophotometer (Model: 170-30, Hitachi, Japan). Inhibition concentration (IC_50_) values were calculated using linear regression analysis and used to indicate antioxidant capacity.

### 2.6 Minerals (calcium (ca), magnesium (Mg), potassium (K))

Exactly, 0.5 g dried plant materials digesting with a mixture 5 mL of (5: 1) of HNO_3_ and HClO_4_. An amount of 5 mL of sample extract and 1 mL of lanthanum chloride was taken in a 50 mL volumetric flask and filled up to 50 mL by distilled water. Atomic absorption spectrophotometer (Model 170-30, Hitachi, Japan) was used at 442.8 nm for Ca, 285.5 nm for Mg, 766.5 nm for K by the methods followed by Sultana et al. [35] and mineral constituents were calculated as percentage.

### 2.7 Disease incidence and shelf life

Fungal disease was identified by acervulus and black colour setae in the infected region by compound microscope (Zeiss Primo Star; Carl Zeiss Ltd.) at the Plant Pathology Lab, BSMRAU and disease incidence (%) at the progressing days after treatment was measured by the following formula (Equation (3)). Besides, the fruit samples were carefully monitored for shelf-life determination. Average shelf life of 5 papayas for a replication of a treatment was calculated to determine the shelf life of that replication of the respective treatment. Shelf life was marked until the fruits lost their edible and marketing quality.

(3) $$\text{Disease}\;\text{Incidence}\;\left(\%\right)=\frac{\text{Number}\;\text{of}\;\text{infected}\;\text{fruits}}{\text{Total}\;\text{number}\;\text{of}\;\text{fruits}}\times 100$$

**Fig. 1:**
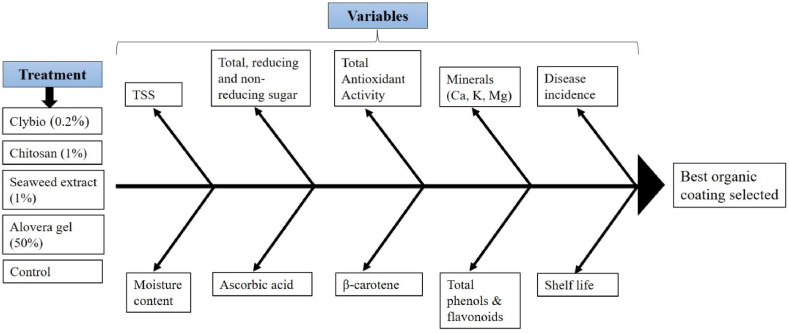
Fish-Bone diagram depicting the various variables that influence the treatment selection.

The treatments and variables studied in the present experiment can be summarized in the following Fish-Bone diagram (Fig. 1.)

### 2.8 Statistical analysis

Data were assessed with descriptive analysis (average ± standard deviation) and analysis of variance (ANOVA) to determine the treatment effect using Statistix-10 computer program. The treatment means were compared by Least Significant Difference (LSD) tests, where p ≤ 0.05 was considered significant. Due to the complete decay of fruit samples, fruit quality traits against some treatments could not be determined after a certain period of storage for statistical comparison. Additionally, correlation matrix, cluster heatmap and principal component analysis (PCA) were employed to sort out the interrelationship among the studied physiochemical and nutritional compositions of papaya as influenced by the varied levels of fruit coatings where the factor loadings and the contributions of each of the studied dependent variables determined using different packages (Agricole, facatominer, factoextra, ggplot2, and corrplot) of R program (version 4.0.2).

## 3 Results

### 3.1 Weight loss and moisture content

A gradual increase in fruit weight loss (%) and a proportional reduction in moisture content (%) with the storage progression were noticed for all the treatments exhibiting significant (p < 0.05) variations among (Table 1). Control fruits, at their shortest storage duration in 6 (six) days after storage (DAS), exhibited significantly maximum weight loss by 30.40 % surpassing the other treatments even at their longest storage duration (12 DAS). Aloevera gel and seaweed extract treated fruits lost 25.04 % and 25.29 % weight, respectively at 9 DAS. Chitosan coating demonstrated superiority over others by minimizing fruit weight loss at all the 3, 6, 9 and 12 DAS (2.01, 6.92, 10.74 and 16.62 %, respectively) which was followed by clybio treatment. Considering the moisture content features, the similar trends of moisture content reduction was noticed compare to the weight loss (%). The rapid moisture reduction from 93.86 % to 86.21 % was observed in control within 6 DAS whereas slow reduction rate was found in chitosan (93.86–88.40 %) from the very day to 12 DAS.

**Table 1:** Effect of various organic coatings on weight loss (%), moisture content (%) and TSS of papaya during storage at room temperature.

Treatment	Weight loss (%)				Moisture content (%)				TSS (Brix%)			
	3 DAS	6 DAS	9 DAS	12 DAS	3 DAS	6 DAS	9 DAS	12 DAS	3 DAS	6 DAS	9 DAS	12 DAS
Control	8.9 ± 2.51^a^	30.40 ± 1.70^a^	–	–	90.04 ± 1.00^b^	86.21 ± 1.10^d^	–	–	8.7 ± 0.35^a^	7.8 ± 0.30^a^	–	–
Chitosan	2.01 ± 1.10^d^	6.92 ± 1.20^d^	11.74 ± 1.00^c^	16.62 ± 0.95^b^	91.96 ± 0.30^a^	90.63 ± 0.33^a^	89 ± 0.50^a^	88.40 ± 0.44^a^	7.2 ± 0.25^b^	7.9 ± 0.27^a^	8.5 ± 0.44^a^	8.7 ± 0.30^a^
Aloevera gel	4.21 ± 1.00^bc^	14.52 ± 0.50^b^	25.29 ± 1.90^a^	–	91.87 ± 0.10^a^	89.78 ± 0.50^ab^	88.54 ± 0.50^ab^	–	7.5 ± 0.30^b^	8 ± 0.31^a^	7.3 ± 0.40^b^	–
Seaweed extract	5.75 ± 0.75^b^	16.31 ± 1.01^b^	25.04 ± 1.30^a^	–	90.77 ± 0.50^b^	89.02 ± 0.40^bc^	87.73 ± 2.00^bc^	–	7.7 ± 0.35^b^	8.2 ± 0.31^a^	8.06 ± 0.35^a^	–
Clybio formulation	2.98 ± 1.00^cd^	9.51 ± 1.52^c^	16.62 ± 0.60^b^	24.74 ± 3.01^a^	90.63 ± 0.40^b^	88.17 ± 0.14^c^	87.30 ± .30^c^	86.35 ± 0.17^b^	7.10 ± 0.29^b^	7.69 ± 0.30^a^	8.4 ± 0.40^a^	8.1 ± 0.21^b^

LSD (0.05)	1.81	2.04	1.62	1.15	0.999	1.006	1.025	0.406	0.556	0.545	0.545	0.632
CV (%)	20.96	7.25	5.68	7.65	0.60	0.62	0.80	0.64	4.32	3.91	3.79	5.38

Values with different superscript letters are significantly different (p < 0.05). Data are presented as mean ± Standard deviation (SD).

### 3.2 Total soluble solid (TSS)

Total soluble contents in papaya increased with advances of days on storage. At three days after storage, maximum TSS was found in control (8.7 %) and minimum TSS was noticed in chitosan (7.2 %) followed by clybio (7.3 %), aloevera (7.5 %) and seaweed (7.7 %). Interestingly the TSS content was increased at 6 DAS except control where it was decreased. After 6 days the control sample lost the shelf life though other treated samples retained the shelf life. Meanwhile, the highest amount of TSS was measured in the chitosan treated sample (8.7 %) and the lowest in clybio (8.1 %) at 12 DAS.

### 3.3 Reducing sugar, non-reducing sugar and total sugar

A gradual increase in reducing sugar was observed for all the treatments in different storage duration (Table 2). The amount of reducing sugar was the highest (22.62 mg/g) in control group at 3 DAS, and reached its peak (23.51 mg/g) before decay compared to other treatments. However, after 6 DAS and onward Chitosan and clybio exhibited a steady increase in reducing sugar to the last with a maximum of 23.78 mg/g and 23.12 mg/g, respectively.

**Table 2:** Effect of various organic coatings on Reducing sugar (mg/g), Non-reducing Sugar (mg/g) and Total Sugar (mg/g) of papaya during storage at room temperature.

Treatment	Reducing sugar (mg/g)				Non-reducing Sugar (mg/g)				Total Sugar (mg/g)			
	3 DAS	6 DAS	9 DAS	12 DAS	3 DAS	6 DAS	9 DAS	12 DAS	3 DAS	6 DAS	9 DAS	12 DAS
Control	22.62 ± 0.15^a^	23.51 ± 0.26^a^	–	–	6.96 ± 0.10^ab^	8.21 ± 0.21^a^	–	–	29.58 ± 0.12^a^	31.72 ± 0.88^a^	–	–
Chitosan	21.32 ± 0.19^b^	22.65 ± 0.20^b^	23.45 ± 0.07^a^	23.78 ± 0.50^a^	6.62 ± 0.50^ab^	7.25 ± 0.25^b^	7.28 ± 0.40^b^	7.97 ± 0.30^b^	27.94 ± 0.40^bc^	29.90 ± 0.32^bc^	30.73 ± 0.62^b^	31.75 ± 0.25^a^
Aloevera	21.22 ± 0.20^b^	22.50 ± 0.50^b^	23.22 ± 0.22^b^	–	6.28 ± 0.35^b^	7.32 ± 0.50^b^	8.25 ± 0.24^a^	–	27.50 ± 0.52^c^	29.82 ± 0.46^c^	31.50 ± 0.55^a^	–
Seaweed extract	22.50 ± 0.25^a^	22.63 ± 0.55^b^	23.36 ± 0.05^ab^	–	5.44 ± 0.56^c^	7.43 ± 0.30^b^	7.90 ± 0.29^a^	–	27.94 ± 0.60^bc^	30.06 ± 0.07^bc^	31.26 ± 0.25^ab^	–
Clybio	21.58 ± 0.34^b^	22.60 ± 0.21^b^	22.75 ± 0.07^c^	23.12 ± 0.20^b^	7.08 ± 0.42^a^	7.37 ± 0.39^b^	7.91 ± 0.20^a^	8.88 ± 0.70^a^	28.66 ± 0.26^b^	29.97 ± 0.18^bc^	30.66 ± 0.37^b^	32 ± 0.40^a^

LSD _(0.05)_	0.432	0.685	0.212	0.438	0.760	0.629	0.477	0.570	0.751	1.10	0.752	0.383
CV (%)	1.09	1.65	0.63	2.56	6.45	4.60	4.18	9.30	3.91	3.79	5.38	3.76

Values with different superscript letters are significantly different (p < 0.05). Data are presented as mean ± Standard deviation (SD).

Similarly, non-reducing sugar had an increasing trend for all the treatment at different course of storage duration. The untreated papaya and alovera gel showed a rapid increase in non-reducing sugar reaching a peak at 6 DAS and 9 DAS with 8.21 mg/g and 8.25 mg/g, respectively than other treatments before decay. On the other hand, Clybio and chitosan treated papaya exhibited a slower rate in the development of non-reducing sugar and in between them clybio retained 13.38 % more non reducing sugar than chitosan at 12 DAS.

Similar increasing trend was also observed for total sugar content. Toal sugar level was recorded highest in control papaya both at 3 DAS and 6 DAS before decay compared to other treatments. Although aloe vera and seaweed extract treated papaya reached the maximum total sugar content at 9 DAS, Clybio and Chitosan treated papaya witnessed a gradual increase from the beginning and reached maximum at 12 DAS, with maximum 32.00 mg/g and 31.75 mg/g, respectively maintaining longer shelf life.

### 3.4 Ascorbic acid and β-carotene content

**Fig. 2:**
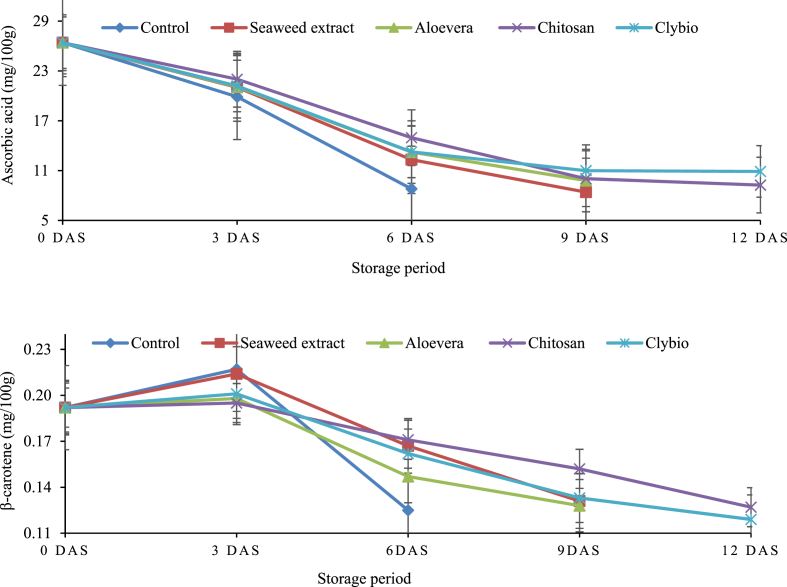
Changes in ascorbic acid and β-Carotene of papaya during storage at room temperature.

All the treatments demonstrated significant (p < 0.05) differences in the degradation pattern of ascorbic acid throughout the storage period (Fig. 2.). At 6 DAS, highest amount of ascorbic acid was found in chitosan (14.96 mg/100g) followed by clybio (13.25 mg/100g), aloevera (13.23 mg/100g) in comparison to control. The papaya treated with clybio had the maximum ascorbic acid retention (11 mg/100 g), followed by chitosan (10.03 mg/100 g) and aloevera gel (9.80 mg/100 g) at 9 DAS. After 12 DAS, clybio had the highest amount (10.9 mg/100g) while chitosan had the lowest amount (9.26 mg/100g) that indicates 6.21 % more retention of ascorbic acid.

Initially, all the treatments showed an increasing trend of β-carotene from 0 DAS to 3 DAS. After 3 DAS β-carotene started to decline and this trend was continued until decay (Fig. 2.). The highest retention of β-carotene was found in chitosan (0.171, 0.152, 0.127 mg/100g) compared to control (0.125 mg/100g) at 6, 9 and 12 DAS, respectively.

### 3.5 Total phenol content (TPC)

**Fig. 3:**
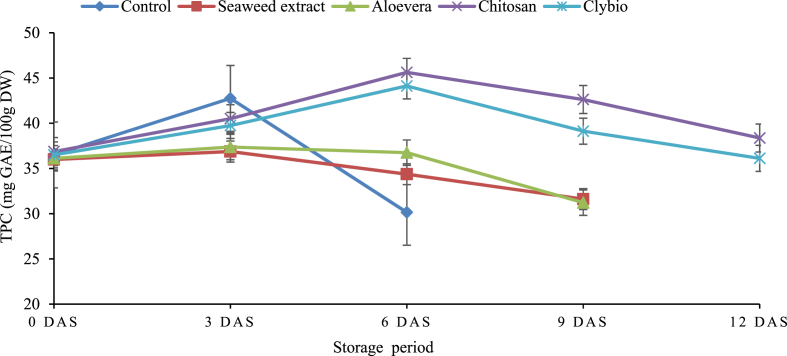
Changes in total phenol content (TPC) of papaya during storage at room temperature.

TPC increased in both treated and untreated papaya from 0 to 3 DAS At 3 DAS, with maximum recoded in control (42.750 mg GAE/100g) and minimum was found in seaweed (36.87 mg GAE/100g) (Fig. 3). Then, chitosan and clybio treated papaya continue increasing trend up to 6 DAS for with maximum amount 45.62 mg GAE/100g, and 44.12 mg GAE/100g, respectively. though other three treatments showed sharp decreasing trend. After 6 DAS, reduction of TPC was found in all treatments that extends until decay. Degradation of TPC was lowest for chitosan than that of other treatments at 6, 9 and 12 DAS.

### 3.6 Total flavonoid content (TFC)

**Fig. 4:**
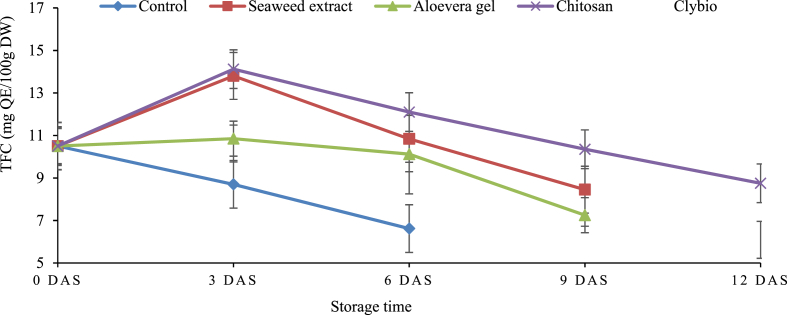
Changes in total flavonoid content (TFC) of papaya during storage at room temperature.

There was significant difference (p < 0.05) on treated and control papaya. Initially, sharp increasing trend was observed for chitosan and sea-weed extract treated papaya while acute decreasing trend was noticed for control treatments. The highest TFC was observed in chitosan treated papaya (14.12 mg QE/100g DW) followed by seaweed (13.80 mg QE/100g DW) and the lowest was in control (8.70 mg QE/100g DW) at 3 DAS. After 3 DAS, TFC tended to show degradation in all treatments. In this case, the highest TFC was found in chitosan coated papaya (12.06 mg QE/100g DW) and the lowest was in control (6.62 mg QE/100g DW) at 6 DAS. Similar trend was observed for all treatments at 9 DAS and 12 DAS (Fig. 4).

### 3.7 Total antioxidant activity

**Fig. 5:**
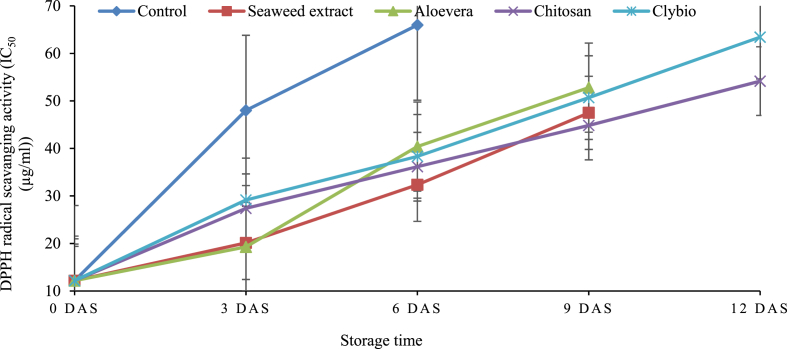
Changes in DPPH radical scavenging activity of papaya during storage at room temperature.

Total Antioxidant Activity (TAA) was measured in terms of DPPH radical scavenging activity (IC_50_). TAA and IC_50_ inversely corelated. TAA was the highest in initial day before applying treatments and later declination of TAA was recorded. The IC_50_ value had increasing trend (TAA had decreasing trend) throughout the storage period for all treatments. The lowest IC_50_ was observed in seaweed (20.12 μg/mL) followed by aloevera (19.28 μg/mL) compared to control (48 μg/mL) at 3 DAS and same as 6 DAS. At 9 DAS and 12 DAS, IC_50_ was minimum in chitosan (44.83 and 54.16 μg/mL) and maximum was found in aloevera (52.79 μg/mL). TAA was found to be comparatively higher in all the coated samples than the control (Fig. 5).

### 3.8 Minerals (ca, mg, K)

Similar increasing trend of Ca content was observed in all treatments throughout the storage period. Initially, there was slight difference in the amount of Ca in different treatments. Nevertheless, Maximum amount of Ca was found in aloevera and seaweed treated papaya while minimum in chitosan at 3 DAS. With advance of storage time, highest Ca was recorded in aloevera at 6 DAS, and in seaweed at 9 DAS whereas chitosan treated papaya had lowest Ca at both 6 and 9 DAS. But at 12 DAS, Ca content reached maximum (0.375 %) in Chitosan treated papaya. Unlike Ca, a declination of Mg was recorded in all treatment. Overall, Mg retention was found consistently maximum (0.223, 0.195, 0.164 and 0.153 %) in clybio treated papaya in all storage interval which was followed by chitosan (0.216, 0.189, 0.158 and 0.136 %). On the other hand, minimum Mg content was recorded in control (0.170 %), aloevera (0.156 %), chitosan (0.144 %) and chitosan (0.123 %) at 3, 6, 9 and 12 DAS, respectively. Among chitosan and clybio, clybio contained 8.31 % more Mg than chitosan at 12 DAS. The amount of K content gradually increased in both treated and untreated papaya throughout the storage duration. There was no significant difference among control, chitosan, seaweed extract except aloevera and clybio treated papaya at 3 DAS. Afterward, Chitosan coated papaya witnessed highest amount of K 1.93 %, 2.00 % and 2.08 % at 6, 9 and 12 DAS. However, the lowest amount of K was observed in aloevera treated papaya (1.75 %), clybio treated papaya (1.85 % and 2.00 %) at 6, 9 and 12 DAS, respectively (Table 3).

**Table 3:** Effect of various organic coatings on weight loss (%), moisture content (%) and TSS of papaya during storage at room temperature.

Treatment	Ca content (%)					Mg content (%)					K content (%)				
	0 DAS	3 DAS	6 DAS	9 DAS	12 DAS	0 DAS	3 DAS	6 DAS	9 DAS	12 DAS	0 DAS	3 DAS	6 DAS	9 DAS	12 DAS
Control	0.234 ± 0.00^a^	0.248 ± 0.50^b^	0.292 ± 0.72^c^	–	–	0.243 ± 0.01^a^	0.170 ± 0.51^c^	0.159 ± 0.86^c^	–	–	1.54 ± 0.01^ab^	1.74 ± 0.02^a^	1.87 ± 0.02^ab^	–	–
Chitosan	0.215 ± 0.02^c^	0.222 ± 0.80^c^	0.275 ± 0.65^d^	0.316 ± 0.60^c^	0.375 ± 0.53^a^	0.246 ± 0.00^a^	0.216 ± 0.01^ab^	0.189 ± 0.52^a^	0.158 ± 0.78^b^	0.136 ± 0.45^b^	1.51 ± 0.04^ab^	1.73 ± 0.03^a^	1.93 ± 0.01^a^	2.00 ± 0.05^a^	2.08 ± 0.06^a^
Aloevera	0.228 ± 0.01^ab^	0.274 ± 0.02^a^	0.328 ± 0.70^a^	0.341 ± 0.76^b^	–	0.227 ± 0.03^c^	0.183 ± 0.36^abc^	0.156 ± 0.01^c^	0.149 ± 0.55^c^	–	1.45 ± 0.01^c^	1.54 ± 0.04^b^	1.75 ± 0.02^c^	1.93 ± 0.08^b^	–
Seaweed extract	0.238 ± 0.00^a^	0.275 ± 0.50^a^	0.315 ± 0.45^b^	0.353 ± 0.90^a^	–	0.230 ± 0.08^bc^	0.172 ± 0.05^bc^	0.174 ± 0.37^b^	0.144 ± 0.53^c^	–	1.56 ± 0.04^a^	1.75 ± 0.02^a^	1.86 ± 0.09^ab^	2.02 ± 0.03^a^	–
Clybio	0.216 ± 0.01^bc^	0.223 ± 0.01^c^	0.268 ± 0.07^d^	0.281 ± 0.66^d^	0.358 ± 0.35^b^	0.240 ± 0.05^ab^	0.223 ± 0.03^a^	0.195 ± 0.47^a^	0.164 ± 0.40^a^	0.153 ± 0.20^a^	1.50 ± 0.02^bc^	1.56 ± 0.03^b^	1.79 ± 0.08^bc^	1.85 ± 0.03^c^	2.00 ± 0.04^b^

LSD _(0.05)_	0	0.013	0.011	0.010	0.078	0.012	0.043	0.013	0.056	0.095	0.055	0.056	0.102	0.069	0.064
CV (%)	0	2.95	2.20	2.18	3.05	2.09	12.50	4.18	3.24	4.07	2.01	1.87	3.05	2.44	4.36

Values with different superscript letters are significantly different (p < 0.05). Data are presented as mean ± Standard deviation (SD).

### 3.9 pH

The initial average value of pH was 4.91, which continued to increase for normal ripening process. There was significant (p < 0.05) variation among different treatment but rapid increment was noticed in control papaya (Table 4). The maximum pH value (5.89) was found in control at 6 DAS where pH of chitosan and clybio treated papaya was only 5.35. At 9 DAS, maximum pH was in aloevera treated papaya compared to all other treatments. However, highest pH was in chitosan treated papaya and lowest was in clybio at 12 DAS.

**Table 4:** Effect of various organic coatings on pH and Disease incidence (%) of papaya during storage at room temperature.

Treatment	pH				Disease incidence (%)			
	3 DAS	6 DAS	9 DAS	12 DAS	3 DAS	6 DAS	9 DAS	12 DAS
Control	5.61 ± 0.10^a^	5.89 ± 0.19^a^	–	–	46.66 ± 11.54^a^	96.67 ± 5.77^a^	–	–
Chitosan	5.01 ± 0.22^c^	5.35 ± 0.13^c^	5.45 ± 0.10^b^	5.69 ± 0.30^a^	0	40 ± 20.00^bc^	60 ± 20.00^ab^	80 ± 20.00^ab^
Aloevera	5.51 ± 0.10^a^	5.59 ± 0.10^b^	5.69 ± 0.29^a^	–	0	20 ± 0.00^c^	80 ± 0.00^a^	100 ± 0.00^a^
Seaweed extract	5.23 ± 0.10^a^	5.45 ± 0.10^bc^	5.49 ± 0.10^b^	–	0	60 ± 20.00^b^	80 ± 5.19^a^	100 ± 0.00^a^
Clybio	5.11 ± 0.18^bc^	5.35 ± 0.15^c^	5.38 ± 0.14^b^	5.41 ± 0.10^b^	0	20 ± 0.00^c^	40 ± 20.00^b^	60 ± 20.00^b^

LSD _(0.05)_	0.181	0.189	0.162	0.115		23.48	23.01	23.12
CV (%)	1.81	1.89	2.03	2.85		27.27	24.33	18.60

Values with different superscript letters are significantly different (p < 0.05). Data are presented as means ± Standard deviation (SD).

### 3.10 Disease incidence (DI)

**Fig. 6:**
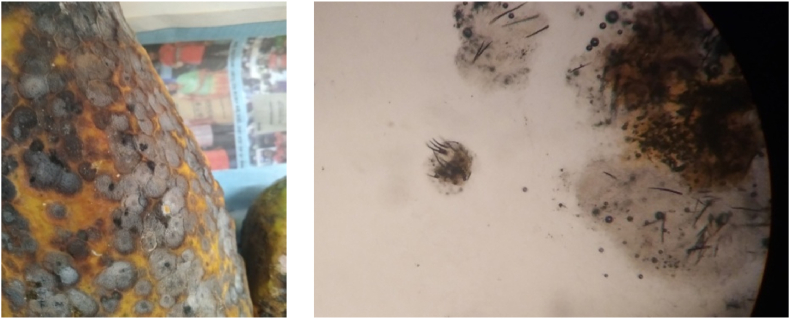
Visual and microscopic view of anthracnose damage and pathogen.

The nonchemical treatments had significant influence on incidence of post-harvest anthracnose disease of papaya caused by *Colletotrichum gloeosporioides* (Fig. 6). Papaya fruits became more susceptible to this pathogen with the increment of storage period and ripening index. Papaya of all treatments except control group was disease free up to 3 days. Afterward, the lowest disease incidence (20 %) was found in aloevera and clybio treated papaya at 6 DAS and highest in control (96.67 %). At 9 and 12 days after storage lowest disease incidence (20–40 % than other treatments) was observed in clybio treated papaya. (Table 4).

### 3.11 Shelf life

**Fig. 7:**
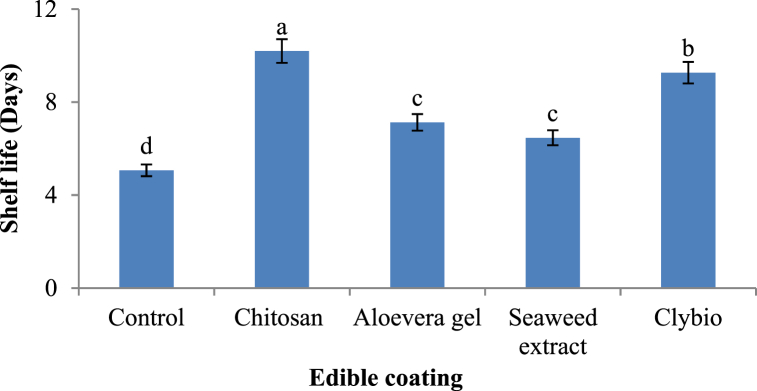
Effect of various organic coatings on shelf life of papaya at room temperature.

**Fig. 8:**
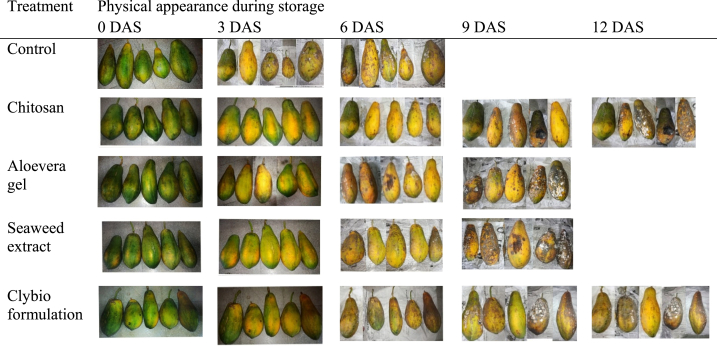
Effect of coatings on physical appearance during storage.

Shelf life of papaya was greatly influenced by the different postharvest treatments and they were statistically significant (p < 0.05) (Fig. 7). The longest shelf-life (10.00[10.20] days) was found in chitosan treated papaya and shortest shelf-life was found in control (5.00[5.06] days). Preceded by that shelf life of papaya in clybio, aloevera and seaweed extract were 9.00[9.26], 7.00[7.13] and 6.00[6.40] days, respectively. Physical appearance of papaya during storage shown in (Fig. 8)

### 3.12 Physio-chemical attributes of papaya at 9DAS

In our study, maximum quality was retained up to 9 DAS for all applied treatments but control papayas were out of consumable and marketable quality after 6 DAS. That's why at 9 DAS datas for control papayas were unavailable. Changing percentage (from initial value to 9 DAS value) for different physio-chemical attributes with treatments application are tried to include in this part.

Improvement was noticed with proceeding of the days of storage for some parameters (Table 5). Initially average value of total soluble solid (TSS) content of fresh sample was 7.08 %. Then it increased gradually in both treated and control papaya. But at peak of the storage at 9 DAS, among treatments the rate of TSS increment was highest in chitosan and clybio and lowest in seaweed treated papaya. Similar case was observed in the change of reducing sugar, non-reducing sugar and total sugar. Rapid increment of reducing sugar, non-reducing sugar and total sugar were observed throughout the storage time for all treatments. Among them the rates of reducing sugar increment were highest in aloevera along with seaweed and chitosan treated papaya compared to clybio at 9 DAS. The rate of change in non-reducing sugar is given in (Table 5) at 9 DAS, where we can see that treated papaya showed positive effect in increment of non-reducing sugar compared to control. However, at the last stage of storage at 9 DAS, the increment percentage of total sugar of treated fruits was about 15.74–18.86 % higher than that of the fresh sample, but there was no significant difference among the treatments. Among treatments highest increment of IC_50_ value was found in seaweed along with clybio treated papaya at 9 DAS that means lowest antioxidant activity. The lowest increment rate was in chitosan treated papaya means highest antioxidant activity. In addition, the highest increment of Ca was found in aloevera (57.70 %) and lowest was in clybio (19.85 %). Disease incidence is properly controlled by clybio at 9 DAS.

**Table 5:** Quality traits improvement of papaya at 9 DAS.

Treatment	TSS	Reducing sugar	Non reducing sugar	Total suagr	TAC (dpph activity)	Ca	K	Disease incidence
Control	0 ± 0.00^c^	0 ± 0.00^c^	0 ± 0.00^c^	0 ± 0.00^b^	0 ± 0.00^d^	0 ± 0.00^e^	0 ± 0.00^c^	93.33 ± 11.54^a^
Aloevera	14.15 ± 1.35^b^	18.31 ± 1.11^ab^	17.06 ± 1.37^ab^	18 ± 3.21^c^	290.61 ± 21.19^bc^	57.70 ± 2.11^a^	29.53 ± 4.21^ab^	80 ± 0.00^a^
Sea weed extract	2.77 ± 1.29^c^	17.67 ± 0.71^ab^	22.34 ± 0.90^a^	18.86 ± 0.36^a^	336.95 ± 13.63^a^	48.54 ± 3.32^b^	33.55 ± 1.04^a^	80 ± 0.00^a^
Chitosan	20.85 ± 2.09^a^	19.08 ± 1.76^a^	9.23 ± 8.30^bc^	15.75 ± 4.54^a^	268.75 ± 23.03^c^	36.79 ± 2.60^c^	32.67 ± 4.45^a^	60 ± 20.00^ab^
Clybio	18.28 ± 0.63^a^	15.23 ± 1.75^b^	17.18 ± 0.04^ab^	15. 74 ± 3.60^a^	316.66 ± 13.26^ab^	19.85 ± 2.83^d^	23.35 ± 3.74^b^	40 ± 20.00^b^
LS	∗∗∗	∗∗∗	∗∗∗	∗∗∗	∗∗∗	∗∗∗	∗∗∗	∗∗

Values with different superscript letters are significantly different (p < 0.01). Data are presented as mean ± Standard deviation (SD).∗∗p < 0.01, ∗∗∗p < 0.00, ∗∗∗ as p < 0.001 that means probability of significance level at 0.01 %.

Quality attributes which trend to degrade during storage are shown in (Table 5). Among them, moisture content decreased continuously due to gradual loss of water by transpiration and other water loss process. At 9 DAS, highest moisture loss rate was found in clybio along with aloevera and seaweed compared to chitosan treated papaya. The highest rate of reduction of the content of ascorbic acid was in aloevera along with sea weed and chitosan compared to clybio. Among treatments, the highest reduction rate of β-Carotene was in seaweed (33.33 %) and lowest was in chitosan (20.83 %). A special case was observed in chitosan and clybio treated papaya where 15.69 % and 7.27 % TPC increased in case of chitosan and clybio treated papaya but 12.06 % TPC in aloevera and 13.46 % in seaweed treated papaya were decreased at 9 DAS. For total flavonoid content (TFC), reduction rate was high in seaweed and clybio treated papaya than fresh sample and lowest was in chitosan treated papaya at 9 DAS (Table 6). Highest reduction of Mg was found in aloevera treated papaya followed by sea weed and chitosan treated papaya compared to clybio at 9 DAS.

**Table 6:** Quality traits deterioration of papaya at 9 DAS.

Treatment	Moisture content	Ascorbic acid	β-Carotene	∗TPC	TFC	Mg
Control	0 ± 0.00^c^	0 ± 0.00^c^	0 ± 0.00^d^	0 b	0 ± 0.00^c^	0 ± 0.00^c^
Aloe vera	6.53 ± 1.06^ab^	68.14 ± 3.79^a^	31.77 ± 0.52^b^	12.06	19.50 ± 0.78^b^	37.27 ± 0.36^a^
Seaweed extract	5.67 ± 0.53^ab^	62.87 ± 3.79^ab^	33.33 ± 0.52^a^	13.46	30.95 ± 0.08^a^	34.06 ± 2.17^ab^
Chitosan	5.18 ± 0.53^b^	62 ± 3.35^ab^	20.83 ± 0.50^c^	15.69	1.14 ± 0.43^c^	35.77 ± 1.10^ab^
Clybio	6.99 ± 0.32^a^	58.33 ± 3.79^b^	30.72 ± 0.55^b^	7.27	26.43 ± 4.13^a^	31.48 ± 2.07^b^
LS	∗∗∗	∗∗∗	∗∗∗		∗∗∗	∗∗∗

Values with different superscript letters are significantly different (p < 0.01). Data are presented as mean ± Standard deviation (SD).∗∗p < 0.01, ∗∗∗p < 0.00, ∗∗∗ as p < 0.001 that means probability of significance level at 0.01 %.

### 3.13 Multivariate analysis

#### 3.13.1 Correlation matrix

**Fig. 9:**
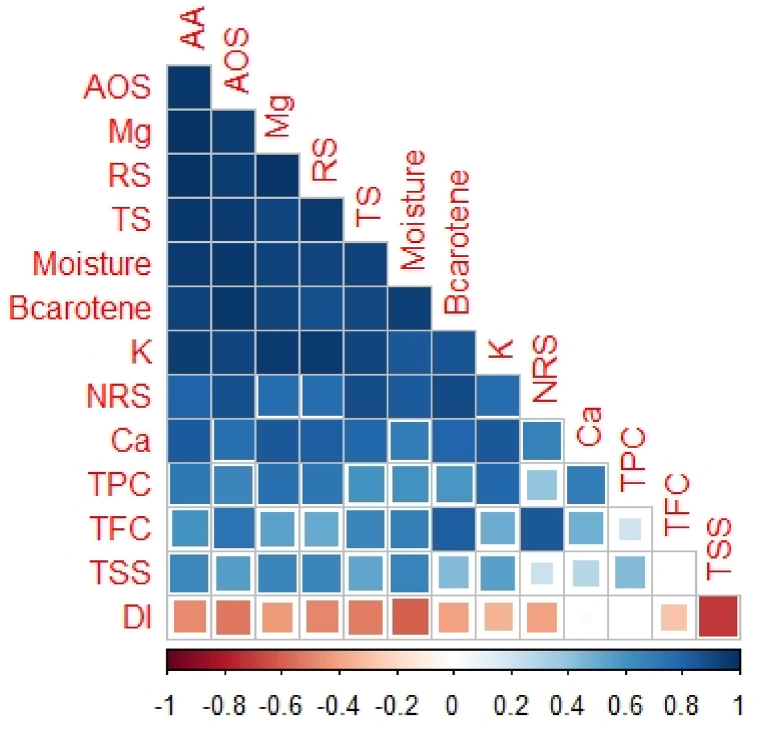
Correlation matrix in between and among changing pattern of parameters studied for papaya quality storage at 9 DAS. Positive correlations are displayed in blue and negative correlations in red color. DI, disease incidence; TFC, total flavonoid content; NRS, non-reducing sugar; TSS, total soluble solid; AOS, Total antioxidants activity; TS, total sugar; AA, ascorbic acid; RS, reducing sugar; TPC, total phenol content.

Pearson's correlation was assessed to prepare a correlation matrix and to detect the interrelationship among positive and negative changing pattern of parameters by different treatments at 9 DAS. Ascorbic acid (AA) positively correlated with all the studied parameters except DI where negative correlation was observed (r = −0.48), where strong correlation with total antioxidants activity (AOS) (r = 0.97). Among sugars, reducing sugar (RS) showed strong positive correlation with Mg (r = 0.97), non-reducing sugar (NRS) with TFC (r = 0.85) where β-Carotene is associated with NRS (r = 0.89) and total sugar (TS) with K (r = 0.91). Moisture content is strongly associated with AOS (r = 0.96). K is strongly associated with Ca (r = 0.85). Except TFC and Ca, all parameters are negatively correlated with DI, where strong negative correlation among TSS and DI (r = −0.70). So, when DI is high, deterioration rate increased in all parameters (Fig. 9)

#### 3.13.2 Heatmap clustering

**Fig. 10:**
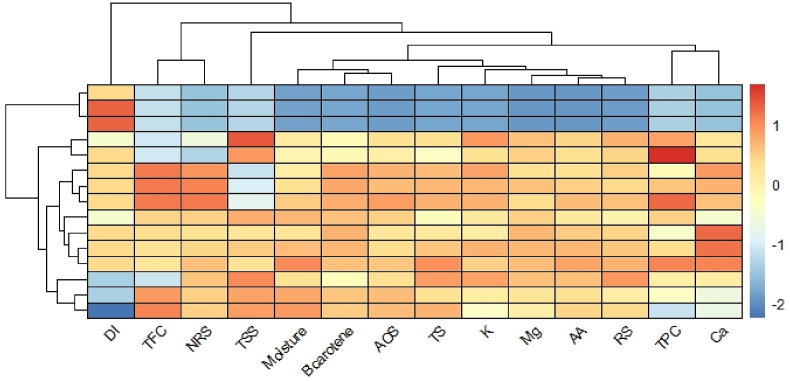
Heatmap representation of changeable parameters at 9 DAS of both treated and untreated papaya with a hierarchical clustering is shown. Blue and red colors represent reduced and increased rate of changes. DI, disease incidence; TFC, total flavonoid content; NRS, non-reducing sugar; TSS, total soluble solid; AOS, Total antioxidants activity; TS, total sugar; AA, ascorbic acid; RS, reducing sugar; TPC, total phenol content.

Changes pattern of parameters at 9 DAS with significant differences among treatments were illustrated through heatmap clustering according to their rate of change from fresh condition. In order to obtain a simplified representation of the quantitative behavior of changeable parameters among treatments, a heatmap clustering was generated (Fig. 10). There are six clusters among parameters. Parameters of each cluster showed similar pattern in increasing or decreasing trend. Among them, cluster 1 (disease incidence), cluster 2 (TFC and NRS), cluster 3 (TSS), cluster 4 (moisture content, β-Carotene and AOS), cluster 5 (TS, K, Mg, AA, RS), cluster 6 (TPC and Ca).

#### 3.13.3 Principal component analysis

**Fig. 11:**
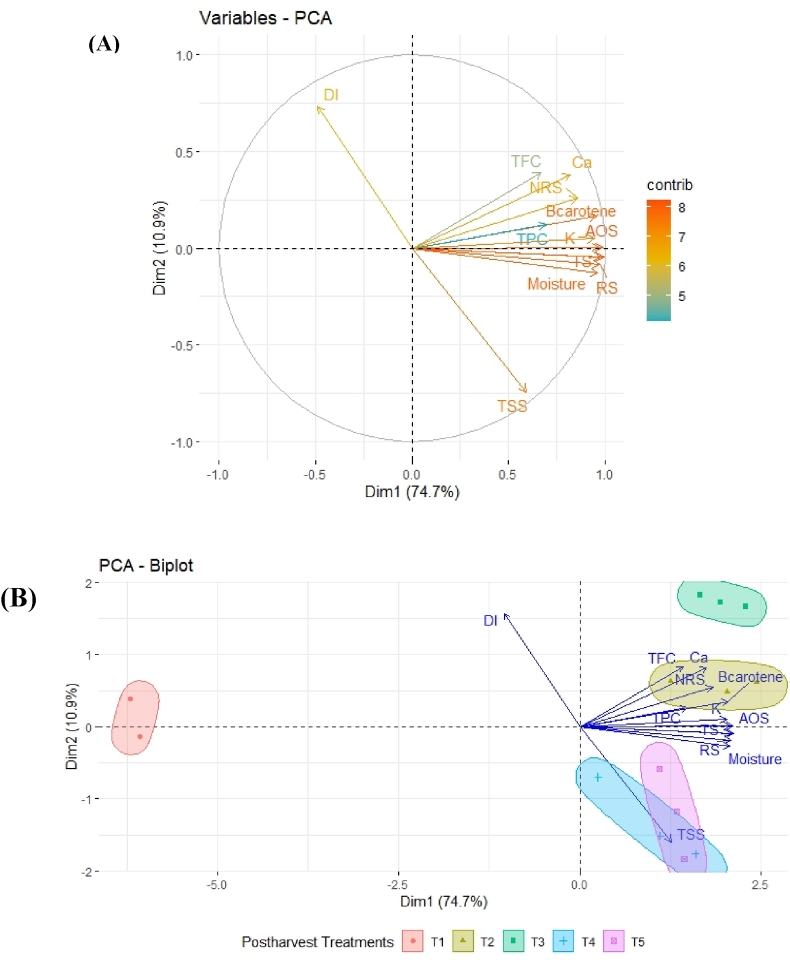
Principal component analysis (PCA) of the changeable parameters studied for postharvest storage of papaya. (A) PCA of the variables showing their major contribution. (B) PCA–biplot analysis representing the performance of changeable parameters effected by different treatments. T_1_, T_2_, T_3_, T_4_ and T_5_ indicate control, aloevera gel, seaweed extract, chitosan and clybio formulation, respectively. DI, disease incidence; TFC, total flavonoid content; NRS, non-reducing sugar; TSS, total soluble solid; AOS, Total antioxidants activity; TS, total sugar; AA, ascorbic acid; RS, reducing sugar; TPC, total phenol content.

In Table 4, Table 5, we can see that postharvest treatments have significant effect on positive and negative changes of physiochemical and nutritional attributes and disease incidence. The average changes of different parameters at 9 DAS of both treated and untreated papaya were correlated with each other (Fig. 11). However, a mere visual inspection cannot properly detect such differences between postharvest treatments. Therefore, all the studied parameters were analyzed using PCA to find out effective postharvest treatment selection. As observed, the two principal components (dimension 1 and dimension 2) explained 85.6 % of the total variations. β-Carotene, AOS, TS, RS, TSS and moisture content were noticed as strong, Ca, NRS, K and DI were noticed as intermediate, and TFC, TPC had low contributions on post-harvest acceptance in papaya at 9 DAS (Fig. 11A). Dim1 explained 74.7 % and Dim2 explained 10.9 % of the total variability among the variables generated by different treatments. Dim1 could be positively associated in a chronological way as follows: AA < RS < TS < moisture content< β-Carotene < K < NRS < Ca < TPC < TFC<, while negatively associated with DI. On the other side, Dim2 could be positively associated chronologically with DI < TFC < Ca < NRS<β-Carotene < TPC < K < TS < AOS, whereas it could be negatively associated with TSS < moisture content < RS < AA < Mg. Among physiological and nutritional quality attributing characters, the most distinct characters were DI and TSS (Fig. 11B) which is associated with maximum quality attributes in storage condition. These two parameters are very important to identify shelf life of the fruit also. And we can see that the important character TSS was controlled by Chitosan and clybio treatment (Fig. 11B). The results of the PCA analysis revealed that among all applied treatment, application of chitosan and clybio had important effects on postharvest storage of papaya. It is clearly understood where green colors indicate the positive association and red colors indicates negative association for both PC1(Dim1) and PC2 (Dim2) (Fig. 11).

## 4 Discussion

Changes in physicochemical attributes of papaya would provide useful information for the evaluation of their storability based on differences in weight loss, nutritional properties, antioxidant activity and disease incidence during storage. This information can create some knowledge for postharvest management of papaya.

Weight loss is an important criterion in quality control as weight loss increased, ﬁrmness decreased and wilting, shriveling or browning increased. The loss of moisture is mainly due to water diffusion through the peel surface which can affect fruit weight loss by respiration and transpiration processes [36]. The positive role of chitosan application in reducing weight loss of papaya could be due to its ability to form a thin semi-permeable film on the outer surface of the fruit which reduces transpiration and affect the gradient of water vapour pressure between fruit and the surrounding air [37]. Similar results of reduced weight loss and delayed ripening have been reported in papaya [8], tomato [38] and cucumber [39].

TSS indicates that fruits are either ripening or at an advanced storage phase. TSS values were slightly increased at initial stage due to conversion of organic acids to sugars by the breakdown of pectin and the transformation of carbohydrates into simple sugars throughout storage due to the metabolic functions of the tissues [40] and then decreased in all other treatment due to the reduction of sugars by respiration process [41] except chitosan. In this study, higher level of total soluble solid in papaya was found in chitosan treated papaya might be due to protective O_2_ barrier reduction of oxygen supply on the fruit surface which inhibited respiration [3]. This result is in agreement with the previous reports in which chitosan coating showed effectively in delaying changes in soluble solids in apple fruit [41].

Sugars can influence the synthesis of specialized ‘sensory’ compounds in fruits [42]. Increment of reducing sugar might be attributed to enzymatic conversion of starch to reducing sugar and also to the conversion of some non-reducing sugar to reducing sugar through the process of inversion [43]. The gradual increase in reducing sugars in chitosan coated papaya as compared to control and other treatment might be due to slow moisture loss through coating and attributed to the slow ripening process [44]. This view is supported by Ref. [45] who showed the positive effect of edible coating on reducing sugar of mangoes and also observed by Ref. [46] in papaya coated with chitosan. The highest amount of non-reducing sugar in clybio until the last day of storage may be due to slow conversion of starch into non-reducing sugar and less bacterial and fungal growth. Similar findings were also reported by Mia et al. [46] in papaya while coated with chitosan. Total sugars of the fruit are considered one of the basic criteria to evaluate fruit ripening. With the passage of time ripening enhances due to ethylene production and enzymatic activity and ultimately total sugars increased [47]. Rapid conversion of starch to total sugar was observed in control papaya might be due to rapid moisture loss [48]. Gradual increment of total sugar in chitosan and clybio treated papaya might be due to slow ripening process and low microbial infection. This view is supported by a report [46] which presented the positive changes of total sugar for chitosan coating in papaya.

In case of secondary metabolites, ascorbic acid affects fruit ripening and stress resistance and plays an important role in quality regulation on postharvest storage [49]. Ascorbic acid content significantly decreased with the increasing of storage period for all treatments and the control. The reduction of ascorbic acid content with storage duration is attributed to respiration (changes in the internal O_2_ and CO_2_ composition of the tissue) and the oxidation of ascorbic acid into dehydro-ascorbic acid by ascorbic acid oxidase enzyme [50]. The chitosan treated papaya followed by clybio treated papaya showed reduced ascorbic acid loss compared to control might be due to the low O_2_ permeability through coatings and delayed deteriorative oxidation reaction. Similar degradation pattern of ascorbic acid was also described by Ref. [38] against chitosan in tomato.

Amount of carotenoids regulated by respiration rates and ethylene production during storage and it is associated with pulp firmness [51]. The content of β-carotene increased from zero to high levels in a few days because of maturation and ripening and then degradation start due to isomerization and oxidation [52]. Carotenoids are highly unsaturated and subjected to Oxidation degradation which is the main cause of major losses of carotenoids is depended upon the availability of oxygen and is stimulated by light, enzymes, metals and co-oxidation by lipid hydro peroxide [53]. In chitosan coated papaya rate of degradation of β-carotene is low might be due to low O_2_ availability through coating and control oxidative degradation that means delay ripening. This type of change of β-carotene was also reported by Refs. [38,53] in fresh cut mango and tomato respectively.

Phenolic components are important secondary metabolites that may directly regulate the color, texture, hardness, and flavor of fruits during storage [54]. The declined trend of TPC and TFC at different days of storage may be due to cell structure breakdown or the activity of yeast and mold [55]. The application of chitosan coating reduced the deterioration of flavonoid by inhibiting the increment of POD activity, which is associated with tissue browning [56]. Chitosan was able to maintain efficient amount of TPC and TFC throughout the storage period until senescence better than other treatments. This finding is in line with the findings of Mendy et al. [57] in papaya and Eshghi et al. [58] in grape for TPC and TFC contents. Pear coated with chitosan also maintained the TFC as reported by Adiletta et al. [59].

IC_50_ value is the concentration of the sample that can scavenge 50 % of DPPH free radical in DPPH free radical scavenging method. As lower IC_50_ value corresponds with a higher antioxidant activity [60]. Antioxidants are effective in scavenging reactive oxygen species (ROS) and minimize deterioration during storage [61]. Edible coatings could modify the internal atmosphere resulting in slowing down the metabolism and delay ripening in fruits [62]. That's why the rate of IC_50_ value increment was low in chitosan coated papaya due to slow ripening process as mentioned in that indicates high antioxidant activity. Chitosan coatings protect the cells against oxidative stress through reactive oxygen species and pathogen attack [63]. Similar findings were described by Hu et al. [61] in sweet cherry coated with chitosan and by Mendy et al. [57] in papaya coated with aloevera.

Minerals are important factor in postharvest quality control of any horticultural crops because they reduce the incidence of internal breakdown disorder by maintaining normal cell-wall structure and by enhancing the resistance against pathogens [64]. In all treated papaya, Ca content was high than control in last consumable stage may be due to reduced water loss, low enzymatic activity and internal breakdown of different components. Mg may maintain cell wall and plasma membrane [65], that's why it could increase the shelf-life. Slow rate of Mg declination of coated papaya might be due to slow rate of metabolic activity of [35]. An identical result was also found in Ref. [38] in tomato. High K content indicated the high-quality control of papaya and it was expressed as shelf-life increment [66]. Similarly [35], reported that tomato fruits contained more Ca and K for chitosan treatment and [66] showed higher K content in banana by aloevera coating and these results are almost at par with the present study

pH value is correlated with acidic value during storage. However, rapid rise of pH within shortest time in control papaya could be due to rapid reduction of organic acid during fast ripening and at the same time slowest rise of pH might be due to reduce the metabolic reactions by creating modified internal atmosphere by coating [67]. Present results are consistent with the findings of Kuwar et al. [67] and Qamar et al. [68] who claimed that edible coating reduced robust increment of pH in strawberry and papaya, respectively.

Anthracnose caused by *Colletotrichum gloeosporioides* is a common postharvest disease of climacteric fruit [69]. Probiotics includes bacterial genera such as *Bifidobacterium, Lactococcus, Carnobacterium, Enterococcus, Streptococcus, Pediococcus, Propionibacterium, Leuconostoc, Bacillus species*, some molds and yeasts which can reduce disease incidence caused by *Colletotrichum* and *Alternaria* [70]. That's why disease incidence was low in clybio treated papaya consisting of 3 probiotics - yeast, *Bacillus natto* and *Lactobacillus.* [71,72] showed effectiveness of probiotics against postharvest diseases of papaya and mango respectively.

Finally, most desirable parameter shelf life indicates the period from harvesting up to the last edible as well as marketable stage [73]. Longest shelf life was found in chitosan coated papaya might be due to slow moisture loss which is associated with cell wall degradation, reduced O_2_ availability and ethylene production that is associated with delayed ripening and reducing decay and retention of secondary compounds which detoxifying the free radicals and ROS [38,73,74]. It ca also be said that, chitosan enhanced papaya's shelf life by creating a physical barrier, reducing microbial load, and minimizing biochemical degradation, ensuring the fruit retains its nutritional quality for extended periods [75,76] as illustrated the similar findings in papaya and banana treated by chitosan.

## 5 Conclusion

The results of this study indicate that papaya treated with chitosan and clybio were the most applicable treatments for retarding repining and extending shelf-life of papaya. Chitosan treated papaya gave the best result than other treatments (except control) up to 9 DAS in terms maintaining moisture content, reducing sugar, β-carotene, total flavonoids content, total antioxidant activity and potassium content. However, clybio formulation proved effectiveness over chitosan up to 12 DAS in some specific parameters such as ascorbic acid, non-reducing sugar, magnesium content and disease incidence. Further research should be conducted at different seasons-including different papaya cultivars, combination of clybio with any other treatments etc. to confirm the precise application of nonchemical substances for postharvest quality improvement of papaya.

## CRediT authorship contribution statement

**Sazia Jahan:** Writing – review & editing, Writing – original draft, Methodology, Investigation. **Joydeb Gomasta:** Writing – review & editing, Writing – original draft, Visualization, Methodology, Formal analysis, Data curation, Conceptualization. **Jahidul Hassan:** Writing – review & editing, Visualization, Supervision, Project administration, Data curation. **Md Habibur Rahman:** Writing – review & editing, Visualization, Supervision. **Md Abdul Kader:** Writing – review & editing, Validation, Data curation. **Emrul Kayesh:** Writing – review & editing, Visualization, Supervision, Methodology, Funding acquisition, Conceptualization.

## Declaration of competing interest

The authors declare that they have no known competing financial interests or personal relationships that could have appeared to influence the work reported in this paper.
